# Emerging molecular testing paradigms in non-small cell lung cancer management—current perspectives and recommendations

**DOI:** 10.1093/oncolo/oyae357

**Published:** 2025-03-24

**Authors:** Frédérique Penault-Llorca, Mark A Socinski

**Affiliations:** Department of Pathology, Centre Jean Perrin, Université Clermont Auvergne, INSERM, U1240 Imagerie Moléculaire et Stratégies Théranostiques, Clermont Ferrand F-63000, France; Oncology and Hematology, AdventHealth Cancer Institute, Orlando, FL 32804, United States

**Keywords:** non-small cell lung carcinoma, biomarkers, targeted therapies, molecular diagnostics, next-generation sequencing, best practices

## Abstract

Advances in molecular testing and precision oncology have transformed the clinical management of lung cancer, especially non-small cell lung cancer, enhancing diagnosis, treatment, and outcomes. Practical guidelines offer insights into selecting appropriate biomarkers and assays, emphasizing the importance of comprehensive testing. However, real-world data reveal the underutilization of biomarker testing and consequently targeted therapies. Molecular testing often occurs late in diagnosis or not at all in clinical practice, leading to delayed or inadequate treatment. Enhancing precision requires adherence to best practices by all health care professionals involved, which can ultimately improve lung cancer patient outcomes. The future of precision oncology for lung cancer will likely involve a more personalized approach, starting increasingly from earlier disease settings, with novel and more complex targeted therapies, immunotherapies, and combination regimens, and relying on liquid biopsies, muti-detection advanced genomic technologies and data integration, with artificial intelligence as a central orchestrator. This review presents the currently known actionable mutations in lung cancer and new upcoming ones that are likely to enter clinical practice soon and provides an overview of established and emerging concepts in testing methodologies. Challenges are discussed and best practice recommendations are made that are relevant today, will continue to be relevant in the future, and are likely to be relevant for other cancer types too.

**Implications for Practice**

Precision medicine in patient care requires specialized knowledge that is best delivered via a well-coordinated and well-educated multidisciplinary team (MDT), up to date with the latest developments in lung biomarker research and testing technology. Molecular testing should be performed in clinical situations where there is evidence that targeting particular molecular alterations makes a clinical difference. Specimen acquisition and processing of tissue should follow well-established standard procedures and protocols, while for genomic testing, next-generation sequencing using multigene panels is highly recommended. Besides methodological aspects, effective collaboration and communication within the MDT are paramount. Patients should be evaluated by MDTs implementing rapid on-site evaluation and reflex testing to improve testing efficiency and reduce the time-to-treatment initiation.

## Introduction

Over the years, lung cancer has served as a prototype disease for medical oncology. Its study has helped to not only understand cancer biology but also importantly to test and develop novel therapies and reinforce paradigms in disease management that are relevant across cancer types. While in the early 20th century lung cancer was considered a rare disease, in the decades that followed its incidence rose sharply. By the middle of the century, single-agent chemotherapy was gradually replaced by combination chemotherapy for both major histologies of lung cancer, non-small cell lung cancer (NSCLC) and small cell lung cancer (SCLC), without much success and the disease became the key example to demonstrate the link between environmental factors, namely tobacco, and cancer. The reality of lung cancer history is that 30 years ago there were editorials written debating whether or not lung cancer was a treatable disease. A landmark meta-analysis by the Non-Small Cell Lung Cancer Collaborative Group (1995) demonstrated that platinum-based chemotherapy can change the natural history of lung cancer by increasing overall survival and was adopted as the standard of care.^1^ Chemotherapy was however a one-size-fits-all approach. The discovery of epidermal growth factor receptor gene (*EGFR*) mutations, first reported in early 2000s, changed that mentality with a number of other mutations/fusions reported subsequently transforming NSCLC into a genomic disease. In parallel, advances in immunotherapy led to such therapies being initially approved in the second-line setting and rapidly moving into the front-line setting.

Even though molecular testing practices are increasing, real-world data reveal underutilization of biomarker testing impacting the broader benefit from targeted therapies, especially in the community setting.^2,3^ A study including patients with newly diagnosed and actively managed advanced NSCLC in the United States in 2019 found that approximately 50% of them did not receive any biomarker testing and among those who did, approximately 30% did not receive appropriate targeted treatments.^4^

Socioeconomic barriers to biomarker testing related to reimbursement, health inequalities and disparity in access are well documented.^5,6^ Logistical issues and practical challenges may occur at any step between presentation, diagnosis, and reporting of results.^7,8^ Waiting for test results and treatment decisions may cause anxiety and stress to patients and their families, while recent studies have shown that at least in NSCLC outcomes depend not only on the time-to-treatment but also on having an optimal pre-treatment assessment and accurately determining tumor characteristics.^9,10^

This review presents a comprehensive but practical view of current molecular testing in lung cancer. It aims to increase awareness of such critical aspects among lung physicians and other healthcare professionals and provide practical advice about how to optimally implement the latest approaches in the day-to-day care of lung cancer patients. It predominantly focuses on NSCLC, as this is most applicable at present, but offers examples and learnings that are relevant to SCLC and potentially to other cancer types too.

## Molecularly guided NSCLC therapies

Approximately 15% of lung cancer is SCLC while NSCLC accounts for the remaining approximately 85% of cases. The latter can be further subdivided into adenocarcinoma and squamous-cell carcinoma, representing 50%-60% and 20%-30% of total NSCLC cases, respectively. The benefits of tyrosine kinase inhibitors (TKIs) for various forms of mutated NSCLC have been remarkable, as for example with osimertinib in first-line therapy of *EGFR*-mutated NSCLC, alectinib and larotrectinib for anaplastic lymphoma kinase gene (*ALK*) and neurotrophic tyrosine receptor kinase (*NTRK*) fusion-positive NSCLC, respectively.^11-14^ Molecularly guided immunotherapy has shown remarkable benefits across NSCLC settings.^15-17^

It is estimated that at diagnosis a total of 50%-60% of patients with advanced NSCLC have an actionable driver alteration, with similar estimates reported for early disease (Table 1).^18-21^ Following treatment, molecular transformation may occur and although the frequency of such molecular alterations during relapse can vary, the most common include *EGFR* mutations, *ALK* rearrangements, fusions involving ROS proto-oncogene 1 receptor tyrosine kinase (*ROS1*) or rearranged during transfection (*RET*), amplification of human epidermal growth factor receptor 2 (*ERBB2*) or mesenchymal-epithelial transition (*MET*), and programmed cell death-ligand (PD-L1) expression.^22-27^

**Table 1.** Actionable mutations in NSCLC.

Gene	Frequency ^a^	ESCAT ^b^	Genetic alteration	Detection method	Sample type	FDA and/or EMA-approved targeted therapies
*ALK*	~5%	IA	Fusions (mutations as mechanism of resistance)	Ventana-D5F3 IHC Break-apart FISH (for rearrangements) RT-PCR detection of specific fusions DNA- or RNA-based NGS	FFPE tumor tissue, liquid biopsy, or cytology specimen	Alectinib, brigatinib, ceritinib, crizotinib, lorlatinib
*BRAF*	~2%	IB	V600E, deletion mutations, fusions, exon 11 mutations	IHC as a screening tool dd-PCR RT-PCR NGS Only for the V600 mutation: Cobas 4800 BRAF V600 Mutation Test and THxID-BRAF kit, monoclonal antibody VE1 the above are only for V600	FFPE tumor tissue, liquid biopsy (blood)	Dabrafenib + trametinib, encorafenib + binimetinib
*EGFR*	~15% ~50-60% Asian	IA	Common: ex19del, L858R	Sanger sequencing Mutation-specific PCR; dd-PCR, NGS	FFPE tumor tissue, plasma	Afatinib, dacomitinib, erlotinib, gefitinib, osimertinib, osimertinib + pemetrexed + platinum chemotherapy, erlotinib + ramucirumab, erlotinib + bevacizumab
	~50-60% of NSCLC cases with acquired resistance to first and second generation EGFR TKIs	IA	Acquired T790M exon 20 (TKI resistance)	Mutation-specific PCR (re-biopsy of the tumor tissue or liquid biopsy)	Plasma (ctDNA)-detection, FFPE tumor tissue	Osimertinib
	10%	IB	“Uncommon” TKI-sensitizing mutations (G719X in exon 18, L861Q in exon 21, S768I in exon 20)	Mutation-specific PCR NGS	FFPE tumor tissue, liquid biopsy (blood)	Afatinib
	4-10%	IIB	Exon 20 insertions	NGS PCR only for specific variants	FFPE tumor tissue, liquid biopsy (blood)	Amivantamab-vmjw, amivantamab-vmjw + carboplatin + pemetrexed
*ERBB2 (HER2)*	2-5%	IIB	Hot spot mutations, amplifications, overexpression	RT-PCR (mutations) IHC FISH (amplifications) NGS (mutations)	FFPE tumor tissue, liquid biopsy (blood)	Fam-trastuzumab deruxtecan-nxki
*KRAS* p.G12C	12% 25-33% all *KRAS* mutations	IIB	Mutation	Mutation-specific dd-PCR PCR pyrosequencing NGS (SiRe® panel)	FFPE tumor tissue, liquid biopsy (blood), plasma (cfDNA)	Sotorasib, adagrasib
*KRAS*	~35%	IIIA	Amplification	Mutation-specific PCR NGS FISH IHC for amplification/overexpression	FFPE tumor tissue, liquid biopsy (blood)	None yet
*MET*	~3%	IB	Exon 14 skipping	IHC FISH Amplicon-based NGS Hybrid capture-based NGS Various modifications of PCR (eg, RT-PCR)	FFPE tumor tissue, liquid biopsy (blood), plasma	Capmatinib, tepotinib
	1-6% of treatment-naïve NSCLC	IIB	Amplifications (de novo or due to acquired resistance on TKIs in patients with various NSCLC mutants)	FISH (MET/CEN7 or MET/CEP7 ratio) NGS assays capable of measuring gene copy number dd-PCR NanoString nCounter	FFPE tumor tissue, liquid biopsy (blood), plasma	None yet
	<0.5%	Not determined	MET fusions	DNA-based NGS RNA-based NGS (amplicon- or hybridization-based) FISH RT-PCR	FFPE tumor tissue, liquid biopsy (blood)	None yet
MSI	0.8%-40%	Unknown	Microsatellite instability, Pattern of hypermutation	IHC PCR (Bethesda, Pentaplex) NGS	FFPE tumor tissue, liquid biopsy (blood)	Pembrolizumab
*NTRK 1/2/3*	0.23%-3%	IC	Fusions	IHC as a screening assay, followed by a validation test DNA- or RNA-based NGS Various modifications of PCR; FISH	FFPE tumor tissue, liquid biopsy (blood)	Entrectinib, larotrectinib
PD-L1	28%: ≥50% TPS 38%: 1–49% TPS 33%: < 1% TPS		Protein expression	IHC (Ventana SP142 and SP263) Dako 22C3 and 28-8 clones	FFPE tumor tissue, liquid biopsy (blood), plasma cytology specimen	Pembrolizumab, nivolumab + ipilimumab, nivolumab + platinum-based chemotherapy, atezolizumab, durvalumab, cemiplimab
*RET*	~1-2%	IC	Rearrangements, fusions	FISH Various modifications of PCR DNA- or RNA-based NGS methods of detection	FFPE tumor tissue, liquid biopsy (blood)	Selpercatinib, pralsetinib
*ROS1*	1-2%	IB	Fusions (mutations as mechanism of resistance), rearrangements	IHC as screening assay followed by a validation test (NGS or FISH) FISH Various modifications of PCR DNA- or RNA-based NGS	FFPE tumor tissue, liquid biopsy (blood)	Crizotinib, entrectinib, repotrectinib
TMB	Data not available	Not determined	High number of coding mutations	NGS	Liquid biopsy (blood)	Pembrolizumab

ALK, anaplastic lymphoma receptor tyrosine kinase; BRAF, B-Raf proto-oncogene, serine/threonine kinase; CEN7, centromere 7; CEP7, centromere of chromosome 7; cfDNA, cell-free DNA; ctDNA, circulating tumor DNA; dd-PCR, droplet digital PCR; EGFR, epidermal growth factor receptor; EMA, European Medicines Agency; ERBB2 (HER2), human epidermal growth factor receptor 2; ESCAT, ESMO Scale of Clinical Actionability for molecular Targets; ex19del, exon 19 deletion; FDA, Food and Drug Administration; FFPE, formalin-fixed paraffin-embedded; FISH, fluorescence in situ hybridization; IHC, immunohistochemistry; KRAS, Kirsten rat sarcoma viral oncogene homolog; MET, mesenchymal-to-epithelial transition factor; MSI, microsatellite instability; NGS, next-generation sequencing; NSCLC, non-small cell lung cancer; NTRK, neurotrophic tyrosine kinase receptor; PCR, polymerase chain reaction; PD-L1, programmed cell death-ligand 1; RET, rearranged during transfection; ROS1, ROS proto-oncogene 1, receptor tyrosine kinase; RT-PCR, reverse-transcriptase PCR; TKI, tyrosine kinase inhibitor; TMB, tumor mutational burden, TPS: tumor proportion score.

^a^Information extracted from.^18-21,96,97^.

^b^The ESMO Scale of Clinical Actionability of Molecular Targets (ESCAT) provides evidence-based criteria to prioritize markers and to select patients for targeted therapies. ESCAT defines six levels of clinical evidence for targets in relation to their implications for patient management, ranging from tier I (ready for implementation in routine clinical decisions) to tier X (lack of evidence for actionability).

These alterations are significant because they can influence treatment decisions and prognosis. For instance, patients with *EGFR* mutations often have targeted therapies available, which can improve outcomes. In addition, there are several other biomarkers whose role in NSCLC is emerging that are being investigated in late-stage clinical trials (Table 2).^28-30^

**Table 2.** Emerging lung cancer biomarkers.

Gene	Frequency ^a^	ESCAT ^b^	Genetic alteration	Detection method	Sample type
*BRCA1/2*	1.2%	IIIA	Mutations	NGS	FFPE tumor tissue, liquid biopsy (blood)
*FGFR1*	9-20%	Not determined	Fusions, amplification	NGS	FFPE tumor tissue, liquid biopsy (blood)
*KEAP1*	15%	Not determined	Mutation	NGS	Liquid biopsy (blood)
*MTAP*	15%	Not determined	Deletion	IHC, FISH	FFPE tumor tissue
*NRG1*	1.7%	IIIB	Fusions	RNA sequencing, FISH, NGS	FFPE tumor tissue
*PIK3CA*	1.2-7%	IIIA	Hotspot mutations	NGS	Plasma (cfDNA), cytology specimen
*STK11/LKB1*	18%	Not determined	Point mutations or deletions	NGS	FFPE tumor tissue, liquid biopsy (blood)
TMB	Data not available	Not determined	High number of coding mutations	NGS	Liquid biopsy (blood)

BRCA, breast cancer gene; CEN7, centromere 7; cfDNA: cell-free DNA; ESCAT, ESMO Scale of Clinical Actionability for molecular Targets; FFPE, formalin-fixed paraffin-embedded; FGFR1, fibroblast growth factor receptor; FISH, fluorescence in situ hybridization; IHC, immunohistochemistry; KEAP1, Kelch-like ECH-associated protein 1; MTAP, methylthioadenosine phosphorylase; NGS, next-generation sequencing; NRG1, neuregulin 1; PIK3CA, phosphatidylinositol-4,5-bisphosphate 3-kinase, catalytic subunit alpha; STK11, serine/threonine kinase 11; TMB, tumor mutational burden.

^a^Information extracted from^28, 29, 30^.

^b^The ESCAT provides evidence-based criteria to prioritize markers and to select patients for targeted therapies. ESCAT defines six levels of clinical evidence for targets in relation to their implications for patient management, ranging from tier I (ready for implementation in routine clinical decisions) to tier X (lack of evidence for actionability).

**Figure 1.**
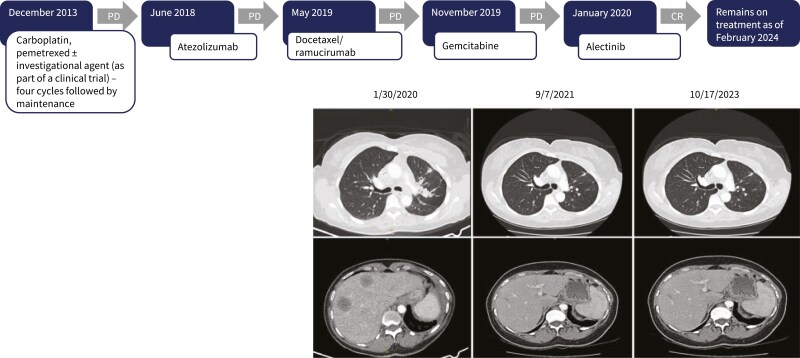
The case is of a 62-year-old female from Trinidad, never smoker who presented with shortness of breath in December 2013. She underwent thoracentesis (left pleural tap) that confirmed adenocarcinoma (thyroid transcription factor-1, TTF-1, +ve). Molecular testing returned negative results for *EGFR*-mutated, *KRAS* wild type, and ALK fusions (the latter performed using FISH). She entered a clinical trial and received four cycles of standard of care plus the investigational agent followed by maintenance, which she continued receiving until June 2018. In the subsequent years, she progressed under other therapies as indicated and in January 2020 her performance status started to decline. At the time, the treatment options considered were palliative care (hospice), another chemotherapy (eg, nab-paclitaxel, irinotecan), or a clinical trial. To aid decision, liquid biopsy for NGS analysis was also performed (preferably chosen over tissue re-biopsy). Plasma-based NGS this time revealed ALK mutation (EML4-ALK fusion) and in February 2020 treatment with alectinib was initiated. The patient’s performance status improved, and she showed complete response (CR) to treatment on follow-up CT scans. She experienced no significant toxicity from alectinib and remains on treatment as of February 2024. Provided courtesy of Dr Tarek Mekhail, AdventHealth Cancer Institute, Florida, USA.

The development of resistance to TKI and immunotherapies is a crucial factor in the management of NSCLC cancer. Although not routine practice, repeated biopsies and testing during disease progression can be used to elucidate the molecular mechanisms underlying the development of resistance mechanisms and guide subsequent therapy, as illustrated in the case study in Figure 1. The expansion of the use of liquid rather than tissue biopsies greatly facilitates this process, where real-time quantitative monitoring of *EGFR* and *ALK* treatment resistance mutations has already been shown to lead to better outcomes.^31-34^

## Aspects to be considered for biomarker testing in NSCLC

**Figure 2.**
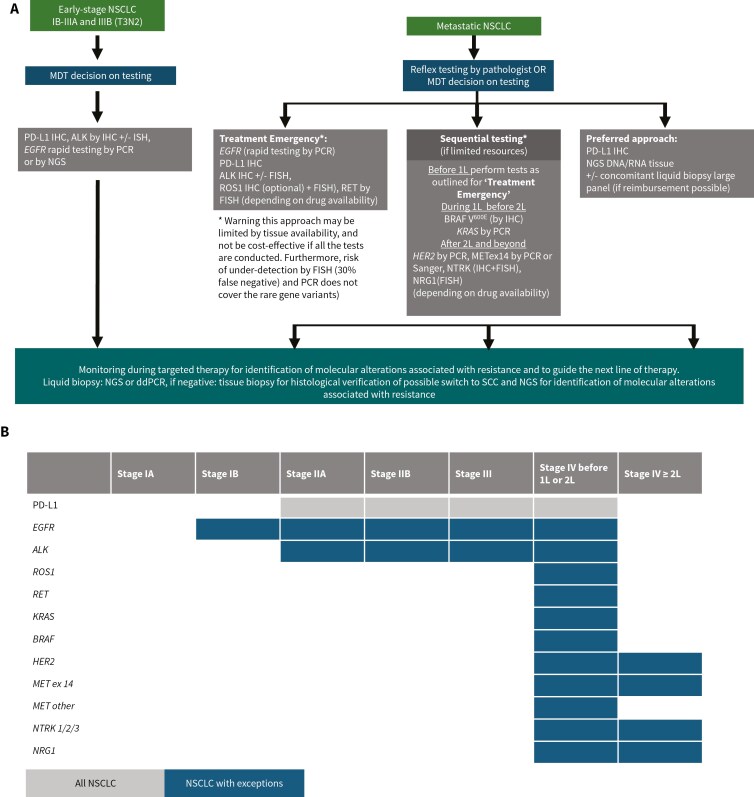
**(A)** A proposed algorithm for minimal molecular testing in NSCLC when comprehensive multipanel molecular testing is not feasible. **(B)** A tabular overview of molecular testing according to disease stage. Although comprehensive molecular testing using broad biomarker panels is the preferred recommendation, in cases where this is not feasible the above proposed minimal testing panels should be considered, depending on the disease stage and particular patient situation. Other specific situations may concern: i) molecular testing for oncogenic drivers is recommended only for non-squamous NSCLC, but the MDT can decide to order testing in certain situations eg, young patients, light smokers, or long-time ex-smokers;^35^ ii) in early-stage disease, it may be reasonable to broaden the search for biomarkers to identify other oncogene-dependent situations (minimally EGFR status, and ALK, ROS1, and RET gene fusions) that show limited or no benefit from immunotherapy, but to date no national or international recommendations include this.^40^ In such cases, initiating targeted therapy before and/or after surgery or radiation may be considered, if no alteration is found in a simple endoscopic or CT biopsy, or in an EBUS sample, but only in the liquid biopsy, and after eliminating possible contamination during the technique and alteration related to clonal hematopoiesis, and the quantification shows a good variant allele frequency. The liquid biopsy result should be taken into account for targeted therapy during the MTB; iii) in the event of disease progression, further immunohistochemical and molecular analyses are indicated. In patients with *EGFR* wild-type tumors, MET expression should be analyzed by IHC, for possible treatment with cytotoxic conjugated antibody (telisotuzumab vedotin) if the tumor shows high membrane MET expression in more than 25% of tumor cells or gene amplification;^41^ iv) in patients receiving anti-EGFR or anti-ALK targeted therapy, it will be necessary to analyze a progressing lesion for histologic transformation to squamous cell or small cell carcinoma and to perform DNA and/or RNA sequencing to identify a molecular mechanism of resistance that may lead to specific treatment: gene amplification or secondary resistance mutations on the oncogene involved, activation of parallel pathways (amplification of *MET* or *ERBB2*, rearrangement of another oncogene, *BRAF* mutation etc.);^42^ v) in large-cell neuroendocrine carcinomas (LCNEC), molecular analysis can distinguish tumors with an adenocarcinoma-like profile (*KRAS*, *KEAP1*, or *STK11* mutations) from those with a small cell carcinoma-like profile (*TP53* mutation and *RB1* deletion or mutation), allowing chemotherapy to be tailored.^43^ Rare cases of targetable oncogenic dependency have been observed in LCNEC.^44^

Clinical practice guidelines, the European Society for Medical Oncology (ESMO), American Society for Clinical Oncology (ASCO), and National Comprehensive Cancer Network (NCCN), offer recommendations how to manage patients with NSCLC emphasizing the importance of appropriate and comprehensive molecular testing (Table 3)^35-39^ Currently, tumor testing at diagnosis and progression on targeted therapy is recommended, while the standard of care mandates testing for all patients with advanced NSCLC and testing to be considered in early-stage NSCLC where therapies targeting EGFR and ALK are available. New therapies and molecular assays are continuously improving, and hence clinical practice recommendations are evolving documents being regularly updated. It should be stressed that that molecular testing at diagnosis and at progression (if feasible) is recommended based on clinical trial data, and that, if feasible, broad molecular profiling should be performed to identify molecular alterations for which there is clinical evidence that targeted or other therapies offer clinical benefit and/or to support early drug access or clinical trials. All expert guidelines support using a broad multi-panel approach is the preferred and most highly recommended option and although recommendations are made mostly based on histopathology, the patient’s clinical situation should also be carefully considered. It follows that the choice of specific treatment should be guided by clinical trial evidence and current recommendations for clinical practice (by ASCO, ESMO, NCCN, or other national professional body). A molecular testing approach with a minimal set of biomarkers that could be followed in cases where comprehensive testing is not feasible according to the clinical situation is proposed in Figure 2.

**Table 3.** Overview of current recommendations for molecular testing in NSCLC.

Molecular testing principles^a^ Molecular testing is mandatory in clinical situations where drugs are approved for routine use. Broader testing may be used to support early drug access or clinical trials. Testing for molecular alterations is important for identification of potentially efficacious targeted therapies, as well as avoidance of therapies unlikely to provide clinical benefit. Administration of therapy should be initiated once molecular testing results are available and based on clinical evidence as appropriate. There may however be exceptions where therapy should be initiated immediately based on patients’ clinical condition. Molecular analyses should be performed in all histological subtypes of non-squamous NSCLC, including adenocarcinomas, NOS (not otherwise specified) carcinomas, large-cell neuroendocrine carcinomas, adeno-squamous carcinomas, and sarcomatoid carcinomas. NSCLC with neuroendocrine features should also be tested, as they are NSCLC. Adequate tissue material for histological diagnosis and molecular testing should be obtained to allow for individual treatment decisions, and re-biopsy should be performed, where possible, when initial sampling is inadequate. Whichever testing modality or methodology is used, it is mandatory that adequate internal and external validation and quality control measures are in place and that laboratories are accredited accordingly, for each test. Molecular testing is recommended in eligible patients with stage IV disease and, for certain biomarkers, in eligible patients with resectable early-stage NSCLC. Testing should be considered in early-stage disease where therapies targeting *EGFR* and ALK are available. Molecular testing for oncogene drivers is recommended in eligible patients with advanced non-squamous-cell carcinoma, although in certain cases it is also recommended for patients with a diagnosis of squamous-cell carcinoma (eg, young patients, light smokers, or long-time ex-smokers). Tissue biopsy is the standard for molecular testing; however, liquid biopsy (ctDNA) may be a complementary approach in some clinical settings, for example, when tissue samples are insufficient or unsuitable/inadequate for biomarker testing, or if re-biopsy cannot be performed safely, when NGS fails, as an alternative to re-biopsy at disease progression or failure of targeted therapy, or potentially to provide a more rapid result. Negative ctDNA tests should be verified by tissue testing, if available. If feasible, testing should be performed via broad, panel-based molecular profiling; if available, multiplex platforms (NGS) for molecular testing are preferable. In some clinical situations, rapid testing may be warranted; nevertheless, it should be followed up with broad-based genomic testing.	
Molecular alteration/test	Recommendations
*EGFR* mutation	*EGFR* FISH or EGFR immunohistochemistry (IHC) has no clinical utility and should not be used *EGFR* mutation test methodology should have adequate coverage of mutations in exons 18-21, including those associated with resistance to some therapies At a minimum, when resources or material are limited, exon 19 deletion, exon 21 L858R point mutation should be determined T790M testing on disease relapse on first- or second-generation EGFR TKIs mandatory Broader liquid biopsy panel to monitor the spectrum of resistance alterations
*ALK* rearrangements	Detection of the *ALK* translocation by FISH is the standard, but IHC with high-performance ALK antibodies and validated assays may be used as a screening approach, or preferably RNA NGS
*ROS1* rearrangements	FISH is the standard or preferably, by RNA NGS
*NTRK* rearrangements	May use IHC for screening but confirmation by molecular testing is mandatory (targeted RT-PCR or preferably RNA NGS)
Other oncogenic drivers	*BRAF* V600 mutation (IHC is available), *MET* exon 14 skipping mutations, *MET* amplifications, *RET* rearrangements, *KRAS* G12C mutations, and *HER2* mutations Tiered testing approaches may be employed ie, certain mutations do not overlap, so testing for one may identify patients who do not benefit from further testing (eg, *KRAS* and *ALK*, *BRAF* p.V600E, EGFR, *MET* ex14 skipping mutations, *RET* rearrangements, and *ROS1* rearrangements Preferably NGS (DNA and RNA) to cover all in one test
PD-L1	IHC must be used If cytology samples are used, individual laboratories should validate their assays in their own cytology preparations against tissue biopsy samples of the same tumor

ALK, anaplastic lymphoma receptor tyrosine kinase; ASCO, American Society for Clinical Oncology; BRAF, B-Raf proto-oncogene, serine/threonine kinase; ctDNA, circulating tumor DNA; EGFR, epidermal growth factor receptor; ESMO, European Society for Medical Oncology; FISH, fluorescence in situ hybridization; HER2, human epidermal growth factor receptor 2; IHC, immunohistochemistry; KRAS, Kirsten rat sarcoma viral oncogene homolog; MET, mesenchymal-to-epithelial transition factor; NCCN, National Comprehensive Cancer Network; NGS: next-generation sequencing; NSCLC, non-small cell lung cancer; NTRK, neurotrophic tyrosine kinase receptor; PD-L1, programmed cell death-ligand 1; RET, rearranged during transfection; ROS1, ROS proto-oncogene 1, receptor tyrosine kinase; RT-PCR, reverse-transcriptase polymerase chain reaction; TKI: tyrosine kinase inhibitor.

^a^As per ASCO, ESMO, and NCCN clinical practice guidelines.^35^

Real-world clinical practice is complex, as local institutional set-up and procedures as well as issues about cost and reimbursement, cannot be ignored. In addition, as mentioned previously, molecular testing is variable and frequently suboptimal, either occurring too late in the diagnostic journey or even not at all, and several challenges may contribute to this (Table 4). In delivering precision medicine-based patient-centered care, effective collaboration and communication are paramount and require several types of expertise and specialized knowledge that is best delivered via a well-coordinated multidisciplinary team (MDT). Logistical issues such as a lack of standardized protocols and operating procedures, together with time pressure or capacity issues hinder efficient communication. The need for sharing and interpreting data in real-time compounds these challenges.^45,46^ Other critical and more practical challenges are discussed below.

**Table 4.** Challenges and best practices of tissue acquisition, tissue quality, biomarker testing, and reporting.

Challenges	Best practices
Tolerability of procedure Patients’ inability to tolerate biopsies hampers tissue collection	Optimize topical anesthesia, anesthetic-led sedation, liquid biopsies as a less invasive procedure
Specimen acquisition; quantity and quality Specimen acquisition procedure can yield limited or inadequate amounts of tissue for comprehensive molecular analysis. More specifically, core needle biopsies may provide insufficient specimen, with low tumor cell number	Use larger gauge needle, if possible and/or apply multiple passes. EBUS-guided ROSE can enhance diagnostic yield and accuracy, may reduce the number of biopsies, and rates of complications Consider liquid biopsies or tumor enrichment MDT/MTB input is helpful in selecting the most appropriate site for biopsy and biological sample for the molecular analysis, for example, histologic sample or liquid biopsy, and the molecular profiling, technologies, molecular test to use The ratio of tumor cells to stromal cells should ideally be ≥20%. It is the responsibility of the pathologist to decide whether or not to attempt molecular assessment, as sometimes the technique can be successful even with low cellularity (5%-10%). In the absence of molecular alteration, the conclusion of the molecular report should state that the result is inconclusive due to low cellularity
Specimen processing Potential degradation of genetic material during processing. RNA prone to degradation; high failure rate of RNA extraction from FFPE. Specific issues include sample loss due to extended periods of ischemia and fixation can impact suitability of samples; use of single cassette can contribute to tissue depletion, necrotic areas may be incompatible with test being used (PCR, NGS; 10%-12% tumor nuclei are required for NGS)	Optimize and standardize operating procedures & ensure staff is trained sufficiently Optimize tissue usage from small samples by dividing biopsies into multiple blocks per test, perform diagnostic IHC upfront, and minimize cutting sessions to reduce tissue waste (limit IHC to necessary tests only) Minimize formalin fixation time to limit nucleic acid damage Minimize cold ischemia to less than 30 minutes for surgical specimens, and immediate fixation for biopsy and cytology Divide tissues into more than one cassette Microdissections may increase viable tumor fraction
Testing methodology and platform diversity Multiple testing options/assays and platforms are available from different vendors, each with specific sample requirements that adds complexity and may lead to confusion and disorganization, ultimately affecting laboratory workflow, efficiency and potentially resulting in longer turnaround times. Multigene NGS panels, more specifically, may identify many genetic variants, some with uncertain clinical relevance, necessitating additional investigation	Optimize and standardize operating procedures & ensure staff is trained sufficiently Standardize requests using a few testing platforms, centralize coordination for efficient sample transport, unify testing processes, and integrate results into Electronic Health Records Initiate reflex testing Combined DNA/RNA NGS is reliable and efficient for comprehensive detection of all approved and emerging biomarkers and is more cost-effective
Reporting Lack of experience in reading genomic reports, especially muti-gene NGS panel reports, and determining clinical relevance The increasing number of biomarkers and treatment options in NSCLC add to the complexity	Standardized molecular genomic report Provide training to enhance molecular genomic report interpretation skills Continuously consult with the latest clinical practice guidelines (incl. ESMO, ASCO, NCCN etc.). MDT/MTB should be advised in the interpretation of complex genetic information. Introduce a genomic coordinator role (or patient navigator) for streamlined coordination
Tumor biology Tumor clonal evolution, resistance, and intra-, or inter-tumor heterogeneity (genetic and molecular) may lead to biomarker discordance among tumor sites and between primary tumor and metastatic site	Consider multiple site sampling; tissue biopsy, cytological samples, and liquid biopsies; particularly in cases involving multiple small primary tumors or metastases
Data collection/collation Incompatibility between Laboratory Information System and Electronic Health Record systems may lead to operational challenges, errors, and inefficiencies	Implement checklists to streamline data capture and reporting Enhance readability and searchability of reports within electronic systems
Quality assessment and control Ensuring consistent and accurate interpretation of results	Perform thorough internal and external validation
Guidance and standardization Limited guidelines for respiratory physicians	Develop/implement best practice guidelines on specimen acquisition for pulmonologist and pathologist (defining critical parameters such as IHC limits, sample marking, and maximizing tissue use, etc.)
Collaboration and communication Diversity in composition and expertise often may affect effective communication and collaboration with the multidisciplinary team/molecular tumor board (MDT/MTB)	Establish clear roles and responsibilities and routes of communication Introduce a genomic coordinator role (or patient navigator) for streamlined coordination Foster a culture of continuous feedback and follow-up to enhance the precision of medical procedures

ASCO, American Society for Clinical Oncology; EBUS, endobronchial ultrasound; ESMO, European Society for Medical Oncology; FFPE, formalin-fixed paraffin-embedded; IHC, immunohistochemistry; MDT, multidisciplinary team; MTB, molecular tumor board; NCCN, National Comprehensive Cancer Network; NGS, next-generation sequencing; NSCLC: non-small cell lung cancer; PCR, polymerase chain reaction; ROSE: rapid on-site evaluation.

### Specimen acquisition

Any method that provides high-quality tissue for diagnosis, staging, and biomarker testing, while posing minimal risk to the patient, may be considered suitable for tissue acquisition.^47^ In diagnosing lung cancer, the most appropriate biopsy site is determined by a team of specialists, including respiratory physicians, pathologists, oncologists, and surgeons. Bronchoscopy, with or without endobronchial ultrasound (EBUS), surgical resections, percutaneous transthoracic core needle biopsy (CNB), or pleural or lymph node biopsy procedures may all be used to collect lung tissue. Cytology-type samples collected via bronchial brushings and washings, percutaneous transthoracic fine needle aspiration (TFNA) or pleural fluid lymph node needle aspirates of accessible metastases may also be considered.^47^

The choice of biopsy needles and number of passes vary, depending on the risk-benefit and tissue requirements, with some experts recommending conducting as many passes as possible to enhance tissue yield. For example, EBUS-guided transbronchial needle aspiration employs needles ranging from 19 to 22 gauge, with three to five passes typically performed. TFNA can use up to 25-gauge needles and transthoracic CNB typically involves needles up to 20 gauge. TFNA without CNB involves multiple passes sufficient to obtain a tissue block for analysis.^48^

### Specimen processing

Once the tissue sample is collected, it is important to use an appropriate and optimal fixative, fixation procedure and duration, to minimize tissue, protein and nucleic acid damage. Neutral-buffered formalin (10%) is the most-used fixative and recommended fixation times are 6-12 hours for small biopsy specimens, and 8-19 hours for larger ones.^48^ Extraction of DNA for molecular-based procedures may be performed using formalin-fixed, paraffin-embedded specimens (FFPE) or fresh frozen, or alcohol-fixed specimens. Usually, 10 unstained slides, each 4- to 5-µm thick, with a surface area between 5 and 25 mm², containing at least 20% tumor nuclei are required. Macro- or micro-dissection is recommended to maximize tumor DNA content and to achieve the ≥20% threshold number of nuclei required.^48,49^ It should be noted that cytology samples typically have lower cellularity, limiting their utility for extensive molecular analysis.^50^

Traditionally, tumor tissue has been the standard biospecimen for most molecular-based biomarker testing. However, it carries several inherent limitations related to the accessibility of the biopsy locations and inadequate tissue for molecular testing and tumor heterogeneity, particularly in patients progressing on treatment. Immunohistochemistry and fluorescence in situ hybridization are not suitable for ctDNA evaluation. Increasingly, liquid biopsy offers a more practical alternative, in the diagnostic setting if tumor tissue testing is not possible and more often at the time of progression or metastasis when tissue may not be accessible. NSCLC has been the main test area for establishing this sampling method for examining tumor-derived alterations.^51^ Liquid biopsy refers to the procedure of collecting blood, from which molecular components can be isolated and examined. In lung cancer, liquid biopsies are most frequently used to isolate circulating tumor DNA (ctDNA). This in turn can be assessed for gene mutations. The first liquid biopsy test ever approved was the cobas EGFR Mutation Test v2 in 2016, which uses plasma specimens as a companion diagnostic test for the detection of exon 19 deletions or exon 21 substitution mutations in the *EGFR* gene to guide therapy with anti-EGFR TKIs, and more sensitive tests have become available since.^52^ Liquid biopsy is not invasive, and bypasses issues related to tumor heterogeneity.^51,53^ Thus having the potential to be used for real-time detection and monitoring of lung cancer disease progression.^54^ It does have limitations however, namely the risk of false negatives (up to 30%) and detection of non-tumor variants, either because the tumor foci have very low shedding and/or because the technique used is not sensitive enough (dd-PCR and NGS preferred) or specific enough (identification of fusions and CNVs).^35^ Due to these reasons and the lack of relevant standards for ctDNA testing, at present, clinical practice guidelines do not recommend using plasma ctDNA testing for NSCLC diagnosis and provide no strict recommendations related to testing during disease progression.^35-39^ It is, however, remarked that upon disease progression, plasma or tissue-based testing should be considered using broad molecular profiling to identify genomic resistance mechanisms. In this case, if plasma-based testing is negative, the panel strongly recommends tissue-based testing with re-biopsy material.

### Genomic testing methodologies

Although genomic testing is sometimes performed as a sequential series of single-gene tests, a more comprehensive multigene testing approach is highly recommended, especially next-generation sequencing (NGS). Tests that directly or indirectly assess single genes include immunohistochemistry (IHC), fluorescence in situ hybridization (FISH), and polymerase chain reaction (PCR), reverse-transcriptase PCR (RT-PCR), or digital droplet-PCR (dd-PCR), a more recent ultra-sensitive method of quantification of targeted mutation.^55,56^

IHC can detect changes at the protein level arising from gene amplifications, as well as from specific DNA rearrangements or point mutations, such as is the case for B-Raf proto-oncogene, serine/threonine kinase gene (*BRAF*) V600E point mutation, *ELM4-ALK*, *NTRK*, and *ROS1* translocations.^57^ IHC is also used to assess PD-L1 expression to guide immunotherapy decisions (a minimum of 100 viable tumor viable cells).^36^ FISH has long been considered the gold standard technique for routine detection of gene amplifications (eg, *MET* and *ERBB2*), especially when IHC results are inconclusive, as well as for identifying DNA rearrangements, such as with *ALK, NTRK,* rearranged during transfection gene (*RET*) and *ROS1* but serial IHC and FISH testing are tissue-consuming and ultimately expensive.^55^

NGS, if available, should be preferable to individual single-gene tests to ensure thorough evaluation for multiple biomarkers.^58,59^ Polymerase chain reaction tests have different levels of sensitivity, with dd-PCR (digital droplet-PCR) being the most sensitive. PCR tests allow a certain level of multiplexing but are limited by the number of potentially targetable mutations in thoracic oncology and can only detect predetermined mutations. Comprehensive NGS panels can detect various genetic alterations, including fusions, CNVs, single nucleotide variants, and small insertions and deletions and may allow the evaluation of tumor mutational burden. If NGS is available, only PD-L1 remains to be assessed using IHC, while all other biomarkers can be assessed with NGS. ALK IHC could also be retained, with FISH control only when rapid results are necessary, in cases having a 2+ score.

It should be noted that not all NGS assays are identical.^60^ There are differences in the type and breadth of biomarkers they can detect and levels of detection, the specific enrichment methods used (especially for targeted assays), tissue requirements for testing, and importantly the associated costs. In most cases, tissue specimens collected from patients with advanced lung cancer have a low tumor cell content and diagnosis often relies on small bronchial or transparietal biopsies. This may pose a challenge for molecular testing, depending on the test platform used.^60^ Interestingly, a high success rate for reporting five or more biomarkers on CNBs and fine needle aspirations has been reported in one study of 1402 NSCLC small samples (10% FNA, 70% core needle biopsies) submitted for clinical NGS testing.^61^

Ideally, NGS testing should cover both DNA and RNA, as assessing DNA alone does not accurately capture all gene fusions (essential for ALK, NTRK, RET, ROS1).^62,63^ Furthermore, comprehensive genomic profiling may provide additional data on molecular alterations beyond established actionable biomarkers.^59^

Two types of NGS assays are commonly used for molecular testing: amplicon-based and hybrid capture-based NGS.^64^ Amplicon-based NGS uses multiple primers for direct genomic region amplification. It shows high analytical sensitivity for specific targets and performs efficiently with limited material. However, it is not as reliable in detecting fusions or copy number alterations. In addition, its multiplex design often limits the number of genes and regions they can cover. Consequently, amplicon-based NGS is mostly used for small gene panels focusing on clinical hotspots or selected areas of interest.

Hybrid capture-based NGS uses hybridization techniques to capture extensive genomic regions, enabling a more comprehensive assessment of mutations, copy number variations (CNVs), and gene arrangements (pre-specified in the panel design). Although typically more complex, hybrid capture-based NGS often has longer turnaround times compared to amplicon-based panels, although it is preferrable for upfront comprehensive testing, if limited sample availability prohibits conducting multiple tests.^47^ Recent ultrafast NGS solutions deliver results for the main actionable targets in an average of 18.3 hours.^65^ Such fully automated solutions could optimize patient care by significantly shortening the time needed to deliver results.

### Staying up to date and interpretation of results

The rapid advancements in the state-of-the art in biomarker research and technology can create gaps in knowledge among healthcare practitioners. Molecular testing and interpreting genomic reports are becoming increasingly complex and health professionals may find it difficult to understand how to correctly read or interpret the results.^66,67^ A recent survey found that oncologists feel less confident when dealing with multigene panel results compared to single-gene tests.^68^ Clinical practice guidelines for the management of lung cancer include guidance on how to perform molecular testing and several professional bodies (ESMO, ASCO, NCCN) have also issued additional specific guidance on how to select patients, perform procedures, or interpret molecular test results.^38,59,69^

## Practical advice for biomarker testing in NSCLC

### Patients should be evaluated by an MDT

Collaborative discussions among oncologists, respiratory physicians, interventional/thoracic radiologists, thoracic surgeons, nurses, pathologists, and/or molecular biologists within MDT are vital throughout the processes involved, from tissue acquisition to testing and interpreting the results.^70,71^ Molecular tumor boards (MTB) play a crucial role in coordinating care and enhancing awareness among the MDT.^72^ This enables informed decisions about patient treatment options and overall leads to improvements in the quality of care and optimization of molecular testing processes. Coordinated testing avoids unnecessary and redundant procedures, and therefore also decreases associated costs.

It is important that electronic health records are accessible and securely shared among all healthcare professionals involved in the patient’s treatment. Information about previous tests results and treatments equips the team with the necessary knowledge to make informed decisions. The inclusion of patient navigators should be considered to act as facilitators, following up on MDT molecular testing decisions and ensuring cross-team alignment.^73^

### ROSE should be applied during specimen acquisition, if possible

Rapid on-site evaluation (ROSE) should be considered as the approach for assessing the adequacy of the specimen effectively. It involves the respiratory physician obtaining a tissue specimen, which is then mounted on a slide by a cytotechnologist and examined by a cytopathologist, who promptly provides immediate feedback on the suitability of the sample for molecular testing.^74^ Implementing ROSE has been shown to improve diagnostic yield, reduce the number of biopsy sites and complications.^75^ It is however a more costly approach, and difficult to implement if there is a shortage of staff and/or the necessary medical expertise. In its absence, a visual inspection of the adequacy of the tissue sample is still valuable. Alternatively, the possibility of using a liquid biopsy, together with tumor cell enrichment techniques to maximize the specimen utility, should be considered.^49,76^ The key limitation to this double evaluation is financial.

### Institutes should implement reflex testing

In de novo advanced NSCLC, reflex testing should be initiated by pathologists immediately after histological diagnosis, eliminating waiting time for physicians to order molecular tests. This proactive approach significantly reduces the time-to-treatment initiation compared to on-demand testing, ensuring faster interventions.^77,78^ Reflex testing not only optimizes tissue usage by analyzing all biomarkers at once but also enhances testing rates and increases the number of patients tested by enabling rapid and standardized ordering of biomarker tests. It was recommended that reflex testing is governed by a protocol defined by the MDT and that the MDT itself should engage with policy makers to establish and maintain the reflex testing protocol.^79^

### Establishing formal platforms for biomarker education

Continued education is essential for healthcare professionals to remain updated in the rapidly evolving field of biomarkers and molecular testing.^80^ Encouraging attendance at conferences, active participation in continuing medical education, journal clubs, and collaborative platforms like MDTs and tumor boards is crucial. Inconsistencies in the use of terminologies related to biomarker testing have been observed, posing risks of miscommunication with potentially other detrimental consequences. The Oncology Nursing Society, for example, has established the Genomics and Precision Oncology Learning Library, providing information on standardized terminology for improved communication.^80^ As mentioned above, professional bodies such as ESMO and ASCO are offering guidance through their clinical practice guidelines but also ad hoc publications and dedicated training workshops.

## New directions and conclusions

While routine molecular testing has not been required for stages I-III NSCLC for many years, this is changing fast. Immunotherapies and more recently targeted therapies are becoming established in non-metastatic disease.^81^ Osimertinib significantly reduces the risk of disease recurrence in the adjuvant setting for patients with stage IB-IIIA *EGFR* mutation-positive NSCLC following complete tumor resection.^82,83^ This underscores the role of molecular testing in early-stage NSCLC, as selection of patients for adjuvant treatment relies on the presence of an EGFR-TKI sensitizing mutation. Furthermore, in the early stages, the prescription of immunotherapy, based on PD-L1 expression levels, is approved for eligible patients pending the absence of *EGFR*-activating mutations and ALK gene fusions.

Encouraging data continue beyond *EGFR-*mutated NSLC. Practice-changing findings have been recently reported from the Phase III ALINA trial of adjuvant alectinib in patients with completely resected *ALK*-positive NSCLC. Alectinib is the first ALK inhibitor to significantly improve disease-free survival across disease stages.^84^ The phase III LIBRETTO-431 trial in RET fusion-positive advanced or metastatic NSCLC in the first line, comparing selpercatinib to control treatment consisting of platinum-based chemotherapy with or without pembrolizumab at investigator’s discretion, reported significant prolongation of median progression-free survival in the selpercatinib group and prolongation in the time to central nervous system progression versus control.^85^ The approval of the antibody-drug conjugate (ADC) fam-trastuzumab deruxtecan-nxki (Enhertu) for the treatment of patients with advanced *HER2*-mutated NSCLC has heralded a new era in lung cancer therapy.^86,87^ Several ADCs and bispecific antibodies are in clinical trials in lung cancer.^87-89^

Multiomic sequencing within oncogenic driver subgroups, such as whole exome, genome, transcriptome, and proteome sequencing, as well as high-throughput functional genomic sequencing and spatial profiling is likely to reveal molecular features associated with response and resistance to therapies. Greater characterization of the underlying cancer heterogeneity and biology (driver alterations) will allow for the development of therapies with higher selectivity and specificity. In parallel, advances in molecular techniques are continuously enabling higher sensitivity assays, sampling technologies, and validating unconventional cytologic and liquid tissue substrates (such as pleural and cerebrospinal fluids) and allows multiomic approaches.^90^ Liquid biopsy technologies have the potential to identify genetic alterations in the diagnostic process and may enable real-time monitoring of the minimal residual disease. Nevertheless, tissue collection is still necessary to assess the initial diagnosis, to evaluate morphological tumor heterogeneity, a possible change in phenotype in the event of resistance to TKIs (switch to small cell carcinoma), and to perform immunohistochemical tests such as PD-L1.

Artificial intelligence (AI) tools are transformative in many ways. AI algorithms can process extensive biomarker data, identifying patterns, and correlations that might elude human attention.^91,92^ Deep learning-based systems are emerging that have been shown to significantly improve the diagnostic accuracy of clinicians and predict the survival benefit in patients with stage IV NSCLC under treatment with EGFR-targeting therapies and immunotherapies and that can assist pathologists in the detection of lung cancer subtypes or gene mutations.^93^ They have, for example, been trained to predict the probability of mutations in the 10 most frequently mutated genes in lung adenocarcinoma tissue (histopathology) images and have shown improved performance in the evaluation of PD-L1 expression consistent with that of pathologists.^94,95^

The future of precision oncology for lung cancer is becoming even more personalized. Emphasis is moving towards having more complex targeted modalities therapies, immunotherapies, and combination treatments that are gradually being used in earlier treatment lines, faster than before. With advances in molecular techniques, the availability of high sensitivity, multi-detection genomic assays, and the growing recognition for validating unconventional cytologic substrates such as urine, sputum, effusions, and FNA, the role of alternate liquid samples will continue to advance. By integrating diverse patient data, ranging from computed tomography (CT) scans to omics information like DNA, RNA, proteins, and microRNA, Artificial intelligence is transforming patient care and advancing not only diagnostic accuracy but also early cancer detection, prognosis prediction, and treatment evaluation. Fundamentally though, enhancing precision requires adherence to current best practices. The concepts and practical considerations presented in this review are relevant today and are likely to continue to be relevant in the future for any healthcare professional caring for patients with lung cancer, and they may very well be applicable to other cancers too.

## Data Availability

Not applicable.
